# Modeling the Insertion Mechanics of Flexible Neural Probes Coated with Sacrificial Polymers for Optimizing Probe Design

**DOI:** 10.3390/s16030330

**Published:** 2016-03-04

**Authors:** Sagar Singh, Meng-Chen Lo, Vinod B. Damodaran, Hilton M. Kaplan, Joachim Kohn, Jeffrey D. Zahn, David I. Shreiber

**Affiliations:** 1 Department of Biomedical Engineering, The State University of New Jersey, 599 Taylor Rd., Piscataway, NJ 08854, USA; sagarsin@scarletmail.rutgers.edu (S.S.); vetdied@gmail.com (M.-C.L.); jdzahn@rci.rutgers.edu (J.D.Z.); 2 New Jersey Center for Biomaterials, 145 Bevier Rd., Piscataway, NJ 08854, USA; damodaranv@dls.rutgers.edu (V.B.D.); kaplanh@dls.rutgers.edu (H.M.K.); kohn@dls.rutgers.edu (J.K.)

**Keywords:** brain-to-computer interface, neural electrode, probe, finite element model, biomechanics

## Abstract

Single-unit recording neural probes have significant advantages towards improving signal-to-noise ratio and specificity for signal acquisition in brain-to-computer interface devices. Long-term effectiveness is unfortunately limited by the chronic injury response, which has been linked to the mechanical mismatch between rigid probes and compliant brain tissue. Small, flexible microelectrodes may overcome this limitation, but insertion of these probes without buckling requires supporting elements such as a stiff coating with a biodegradable polymer. For these coated probes, there is a design trade-off between the potential for successful insertion into brain tissue and the degree of trauma generated by the insertion. The objective of this study was to develop and validate a finite element model (FEM) to simulate insertion of coated neural probes of varying dimensions and material properties into brain tissue. Simulations were performed to predict the buckling and insertion forces during insertion of coated probes into a tissue phantom with material properties of brain. The simulations were validated with parallel experimental studies where probes were inserted into agarose tissue phantom, *ex vivo* chick embryonic brain tissue, and *ex vivo* rat brain tissue. Experiments were performed with uncoated copper wire and both uncoated and coated SU-8 photoresist and Parylene C probes. Model predictions were found to strongly agree with experimental results (<10% error). The ratio of the predicted buckling force-to-predicted insertion force, where a value greater than one would ideally be expected to result in successful insertion, was plotted against the actual success rate from experiments. A sigmoidal relationship was observed, with a ratio of 1.35 corresponding to equal probability of insertion and failure, and a ratio of 3.5 corresponding to a 100% success rate. This ratio was dubbed the “safety factor”, as it indicated the degree to which the coating should be over-designed to ensure successful insertion. Probability color maps were generated to visually compare the influence of design parameters. Statistical metrics derived from the color maps and multi-variable regression analysis confirmed that coating thickness and probe length were the most important features in influencing insertion potential. The model also revealed the effects of manufacturing flaws on insertion potential.

## 1. Introduction

Brain-to-Computer interface (BCI) devices have been gaining traction towards clinical usage [1,2], particularly for rehabilitation following central nervous system (CNS) injury. BCI devices generally operate by acquiring a volition signal, processing the acquired signal, and translating the processed signal into the operation of an extra-corporeal device. Successful operation of a BCI device is foremost dependent on accurate signal acquisition [3]. The three most common modalities of acquiring volition signal, in order of least-to-most invasive, accurate, and resolved are: (1) Electroencephalograms (EEGs); (2) Electrocorticographs (ECOGs); and (3) Single-Unit Recordings (SURs) from neural probes. Neural probes in particular have been widely used in research as evidenced by the popularity of the Utah Array [4,5] and the Michigan Array [6,7].

However, current neural probes are limited by their inability to maintain signal fidelity for long-term acquisition of SURs. Probe insertion into brain tissue injures cells and surrounding microvasculature. A wound healing response is initiated that includes microglia and astrocyte activation, which can hinder signal acquisition [8,9,10]. Moreover, a chronic response to the implanted probe is also observed. Reactive astrocytosis persists long-term from shear stresses at the probe–tissue interface, which ultimately leads to glial scarring [11,12]. Astrocytes comprising the glial scar encapsulate the recording electrode, which increases the local impedance and limits electrode contact with surrounding neurons. As a result, current probes, which are successful in obtaining accurate signals in the short-term, fail to maintain adequate signal-to-noise ratio in long-term use [13,14,15].

Among the many features believed to influence the magnitude of this foreign body response, probe geometry and material properties are especially relevant from the perspective of probe design. Previous studies have shown that larger probes induce a greater magnitude of primary injury and a greater degree of long-term gliosis [16,17]. The chronic response is also influenced significantly by the probe material properties. Stiffer probes have been linked to more extended and severe chronic responses in comparison to probes that have mechanical properties similar to CNS tissue [10,17,18]. These observations suggest that a smaller, more flexible microprobe will minimize primary trauma and the subsequent chronic response [19].

However, small, flexible probes may be too weak to insert into brain tissue and must be assisted by a stiffer and/or larger device. For example, flexible probes have been inserted through insertion shuttles that confer temporary mechanical strength to the probe. Once the probe is successfully in place, the shuttle is removed surgically. Unfortunately, these shuttles induce significant primary trauma [20].

Another approach to providing temporary mechanical strength is coating or supporting a flexible probe with a sacrificial, protective polymer, which degrades following insertion. Ideal coatings would also degrade rapidly into non-cytotoxic byproducts and leave the intact probe exposed to nearby neurons for signal acquisition as early as possible after implantation. Previous groups have utilized polymer coatings made of nitrocellulose-based materials [21], poly(DL-lactide-co-glycolide) [22], polyethylene glycol (PEG) [11], and tyrosine-based compounds [23]. Each of these has shown promise as a temporary coating due to their biocompatibility and substantially greater stiffness relative to probe stiffness. The tyrosine-based compounds have the added benefit of ultrafast degradation, which removes the insulating coating layer within hours to allow for earlier neural recordings [24]. Given the properties of the coating or supportive material and the properties of the probe, it is possible to design the probe to optimize mechanical performance while minimizing primary trauma. The ideal coating would have the minimum coating thickness necessary to allow successful insertion, which would mitigate tissue trauma during insertion and leave a flexible probe that would minimize interfacial stresses. Given the wide range of probe and coating specifications, experimentally finding an acceptable range of designs that fit these criteria can be a costly and time-consuming task.

Computational modeling can be an effective approach to evaluate the effects of different specifications on probe insertion and mechanical performance in soft tissue. Finite element models that simulate needle insertion into soft tissues have been employed in prosthesis design [25,26,27]. Similar principles have been applied to neural microelectrodes, particularly to understand how probe geometry [15,28] and material properties [29] influence probe–tissue interfacial stresses. Other groups have concentrated on modeling the mechanical trauma exerted on the brain during and post-insertion to ascertain the probe’s “neural kill zone” [13], but none have modeled the effects of design criteria for insertion mechanics, particularly for coated, flexible probes.

We have developed an approach for the reproducible manufacture of polymer-supported microprobes using microfabrication techniques [30]. We first established our approach by fabricating microprobes with SU-8 photoresist, and then adapted the methods to fabricate microprobes from Parylene C, a class VI polymer used in many medical devices that is highly flexible to better match the compliance of brain tissue. Parylene C and SU-8 have similar tensile stiffness, but significantly differ in their flexural stiffness, which dictates the response to the primary loads experienced by the probe during and after insertion [31]. The probes are coated with an ultra-fast degrading tyrosine-based polymer that is rigid during insertion but is hydrolytically degraded within hours following insertion [24]. The goal of this study was to develop and validate a finite element model that predicted coated probe performance during insertion into brain tissue. We first developed finite element simulations to model buckling and penetration into tissue, and validated predictions made by each model by testing varying sizes of copper wire. Next, we compared the model predictions to results from experimental tests of uncoated and coated SU-8 and Parylene C probes to validate the model for softer materials. Finally, we used the model to predict the performance of uncoated and coated Parylene C probes over a range of design specifications, employing a number of metrics to assess which features of the probe and coating design influence insertion and tissue damage, as well as the design regime of coated flexible probes that will insert and minimize insertion trauma.

## 2. Methods

### 2.1. Buckling and Insertion Tests

**Figure 1:**
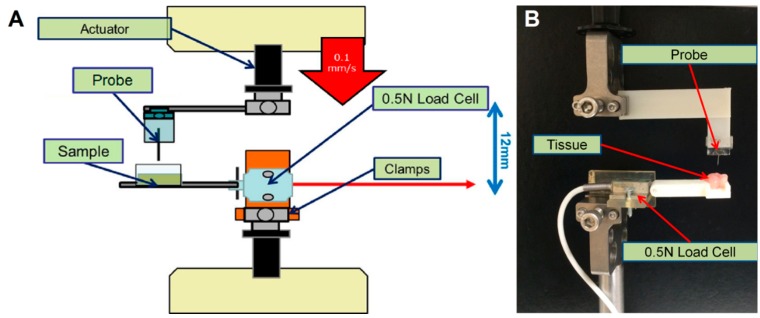
(**A**) Mechanical testing setup to experimentally characterize probe and coating behavior. Probes were fixed on a glass slide and clamped to a rapid-prototype piece, which was clamped to an actuator on a Bose ELF 3200. A 0.5 N load cell was threaded onto a rapid-prototyped piece, which was clamped to the reaction plate of the testing device. A third piece containing a well for the sample (agarose or brain tissue) was pinned to the load cell; (**B**) Zoomed-in image of the fixed probe and sample of embryonic chick brain tissue.

Buckling and insertion testing was performed with an Enduratec ELF 3200 uniaxial mechanical testing device (Bose) (Figure 1). Probes or probe mimics were secured to an actuator, which lowered the probe into an agarose tissue phantom, chick embryonic brain tissue, or adult, rat brain tissue. These tissues or tissue phantoms were set on a rigid surface, which was connected to a 0.5 N cantilever-type load cell (Entran Sensors and Electronics, Fairfield, NJ, USA). For tests with an agarose phantom, a 0.6% by weight solution of low-temperature melting agarose (Sigma-Aldrich, St Louis, MO, USA) was prepared. This concentration of agarose has mechanical properties similar to brain tissue and has previously been used as a tissue surrogate [24,32]. For tests with chick embryonic brain tissue, which provided an inexpensive and convenient source of model tissue, fresh fertilized chicken eggs (Charles River Labs, North Franklin, CT, USA) were incubated for 18 d. The embryo was extracted from the egg, and brain tissue was excised. Finally, for tests with rat brain tissue, rat brains were extracted from female Sprague-Dawley rats (Charles River Labs, North Franklin, CT, USA) following euthanasia via asphyxiation in 100% CO_2_ environment following an approved IUCAC protocol. Preliminary experiments showed that the presence of the dura layer had negligible effect on insertion force and thus was left intact. Brains were transferred to 37 °C saline and used immediately following removal.

Probes or probe mimics were lowered at a rate of 0.1 mm/s into the agarose phantom or brain tissue. A humidifier was used to ensure tissue remained hydrated during testing. The probe either buckled after contact with the tissue/phantom, or it penetrated the tissue/phantom, traveled through the entirety of the tissue or phantom, came in contact with the rigid surface underneath the tissue/phantom, and then buckled. No probes that penetrated the surface of the tissue or phantom failed to reach the rigid underlying surface. The insertion force was defined as the peak force in the recorded profile during penetration of the tissue but before contact with the rigid surface. Buckling force was defined as the peak force generated once the sample came in contact with the rigid surface. In general, forces for probes that buckled during penetration of the agarose phantom and/or brain tissue were below the limit of detection for the transducer (0.1 mN).

The insertion and buckling tests were performed with either uncoated copper wire, coated or uncoated SU-8 probes, or coated or uncoated Parylene C probes. Initial tests were performed with copper wire with the sole intent of providing data to validate simulation results. Three different diameters (320, 750, and 875 μm) of copper wire were first tested with agarose phantoms. A number of SU-8 and Parylene C probes (Table 1) were tested in embryonic chick brain tissue. Eight probes were tested in each cohort, and the number of successful insertions was recorded. Four probes were tested in each brain, with a minimum distance of ~1 mm between insertion locations to prevent the possibility of insertion in damaged tissue.

Finally, select Parylene C and SU-8 probe designs were tested in rat brain tissue (Table 2). We purposely selected a range of probe and coating designs for insertion into rat tissue that were expected to all fail (all fabricated probes fail to insert), all succeed (all fabricated probes successfully insert), or demonstrate a mixture of failure and success (some but not all fabricated probes successfully insert). These expectations were based on model predictions for insertion and buckling forces after validation against the agarose and chick tissue data. Twelve probes were tested in each cohort, and the number of successful insertions was recorded. All tests were completed within an hour post sacrifice to prevent any substantial change to the *ex vivo* brain’s material properties [33]. The minimum distance between insertion locations within the same brain was ~1 mm.

### 2.2. Finite Element Analysis

Probe mechanics were simulated in ABAQUS 6.10 (Simulia). Two models were developed: one to simulate buckling of uncoated and coated probes, and the other to simulate insertion into a tissue phantom.

#### 2.2.1. Probe and Coating

Probes and coatings with varying design parameters (see Table 3 for a full list) were generated and meshed with 8-node, reduced-integration (C3D8R) elements. The probes were completely coupled to the coating by specifying a tied constraint between contacting surfaces. To validate the model, probes were modeled as one of three different materials to match buckling and insertion experiments: copper, SU-8 photoresist, and Parylene C. For each material, we used the flexural modulus. With the exception of the flexural moduli for SU-8 and Parylene C, values for material properties were obtained from the literature: copper (E = 110 GPa, *ρ* = 8960 kg/m^3^, ν = 0.36), SU-8 photoresist (E = 2.4 GPa, *ρ* = 1190 kg/m^3^, ν = 0.32), or Parylene C (E = 5.6 MPa, *ρ* = 1289 kg/m^3^, ν = 0.45) [34,35,36,37,38,39]. The tyrosine polycarbonate coating was modeled using material properties from previous characterizations (E = 1.9 GPa, *ρ* = 1290 kg/m^3^, ν = 0.42) [24].

To determine the flexural modulus experimentally for SU-8 photoresist and Parylene C, strips of varying dimensions for SU-8 (0.5–2 cm × 500 μm × 20 μm), and Parylene C (1–3 cm × 1 cm × 20 μm) were clamped at one end and allowed to deform under their own weight in cantilever bending. Digital microcalipers were used to measure the maximum deflection. Stiffness was calculated by using the beam deflection equation [31]:

$$E=\frac{qL^{4}}{8I\delta_{max}}$$

where *q* is the weight per unit length of the strip; *L* is the length of the strip; *δ_max_* is the maximum deflection of the strip; *I* is the area moment of inertia of the cross-section of the strip; and *E* is the flexural modulus. Images of the bending profile were captured and examined to ensure that appropriate bending behavior was observed. Deflection profiles for lengths of Parylene C and SU-8, with their calculated flexural moduli are provided in Supplementary Figure 1 (Figure S1) and Supplementary Table 1 (Table S1) respectively.

#### 2.2.2. Buckling Model

A linear buckling analysis was performed in ABAQUS to identify the force required to initiate buckling. Contour maps were generated to evaluate force distributions along the probe and coating. The buckling force for each model execution was determined by calculating the mean maximum force of the nodes in the probe and coating.

#### 2.2.3. Insertion Model

**Figure 2:**
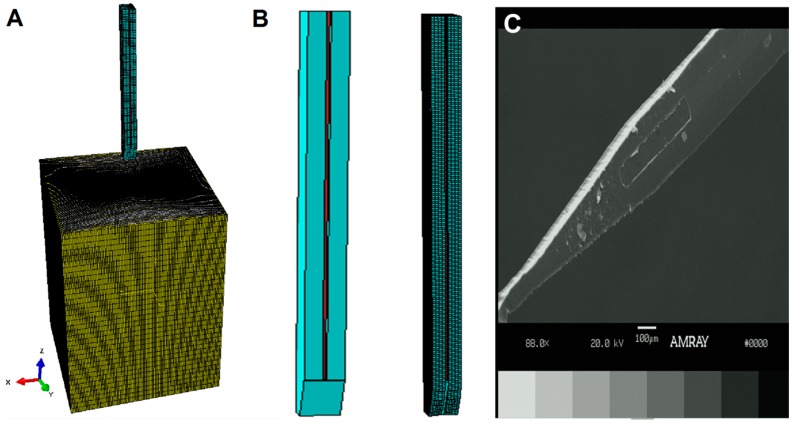

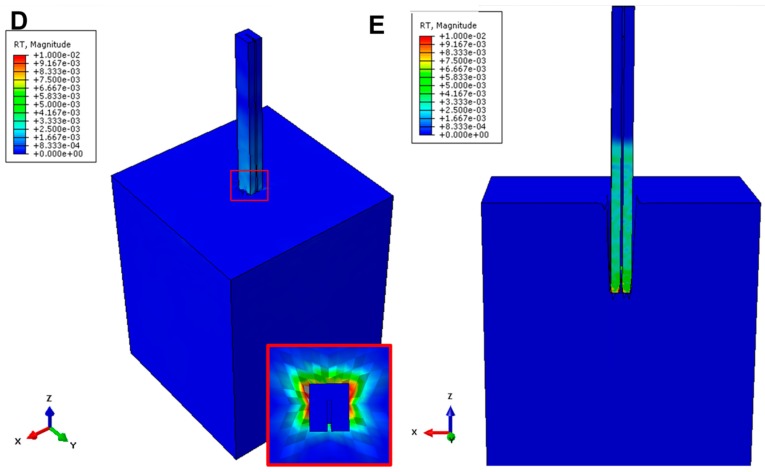
(**A**) Screenshot of Finite Element (FE) model. Stiffness, dimensions, and geometries were varied for the probe (red), beveled coating (light blue), and brain tissue (grey/yellow). The model was executed to simulate insertion of the coated probe into brain tissue. Infinite elements (CIN3D8—yellow) were defined around the edges of the brain instance to reflect the significantly larger size of the brain relative to the coated probe. The bottom of the instance was fixed with a zero-displacement and zero-rotation constraint; (**B**) A close up of the simulated coated probe before and after meshing; (**C**) a scanning electron microscope image of the coated probe; and (**D**) a screenshot of the inserted probe shortly after penetration. Nodes that experienced force at this step had their maximum force averaged to determine insertion force. The zoomed-in region depicted in the red box shows the contour map of strains experienced on the surface of the brain instance. Elements in grey have reached the failure threshold (0.05) and are deleted in the next step. (**E**) Cut-away of the coated probe one second later than shown in (**D**) insertion, showing that only the coating experiences force during insertion.

Probe insertion into brain tissue was modeled with a dynamic, explicit analysis in ABAQUS. Agarose and brain tissue were modeled as hyperelastic materials using Ogden material parameters adapted from the literature [40,41] with C3D8R elements and adaptive meshing. To emulate probe penetration, elements that exceeded a shear strain of 0.05 were deleted from the mesh prior to the next increment in time. This value was selected based on preliminary results of insertion tests, where the threshold value for element deletion in our simulation was modified until predicted insertion forces matched experimental results in insertion tests with the agarose phantom and brain tissue. The influence of this value was evaluated with a sensitivity analysis (described below). Infinite elements were used at the edges of the tissue to simulate the significantly larger size of the tissue with respect to the size of the probe. Coated probes were inserted at a rate of 0.1 mm/s into the substrate. The bottom of the tissue was fixed (Figure 2). The top of the probe and coating was only permitted to move in the z-direction. Similar to the buckling case, contour maps were generated to visualize force distributions along the probe and coating. An average insertion force was calculated as the average peak force for the nodes in the coating and probe identified from force *vs.* time plots.

#### 2.2.4. Sensitivity Analysis

In addition to varying probe geometry, a sensitivity analysis was performed to determine the influence of the parameters used to define material properties and failure thresholds of the hyperelastic brain tissue on the predicted insertion forces. Material properties were adjusted to a maximum of ±10% [42], the coefficient of friction between tissue and coating/probe was varied from 0 to 0.5, and strain thresholds for element deletion were varied from 0.01 (extreme) to 0.25 (conservative) based on information in the literature [43,44,45]. A surface–surface contact algorithm was specified.

To assess sensitivity to coating flaws, a cylindrical void region was introduced in the coating. The radius of the void region was varied by proportion of the coating thickness, from 5% to 100% of the coating thickness. Four coating thicknesses were modeled (50, 75, 100, and 200 µm). The percentage change in insertion and buckling forces relative to simulations with a non-defective coating was calculated.

Each model was executed on a *Lenovo Z580* (Intel^®^ Core™ i5-3210M CPU @ 2.50 GHz; 6 GB RAM) or a *Lenovo Y510* (Intel^®^ Dual Core™ 2.81 GHz; 4 GB RAM). A single design simulation took approximately 7–12 min to execute depending on the size of probe/coating. Simulations were run in parallel. A total of about 28,000 simulations were executed.

### 2.3. Data Interpretation

To provide a performance metric for the simulation results, the ratio of the predicted buckling force to the predicted insertion force was determined for each simulation. We termed this ratio the “safety factor” for that particular design. A probe design would have a safety factor of 1 if the predicted buckling force equaled the predicted insertion force. To assess the influence of each parameter on performance, we designed a graphical user interface in MATLAB to generate safety factor probability maps. Model results for varying parameters (outlined in Table 3, column: “Range modeled”) were tabulated and read by the script. Interpolation methods were used to compute gaps in values, and color maps that plotted two parameters against each other were generated.

In addition to providing a visual assessment of performance, the color maps were also quantitatively assessed using three statistical measures—kurtosis, variance, and skewness—to draw numerical comparisons between pairs of parameters. Kurtosis indicates the “peakedness”, or sharpness of the peak in the distribution. Color maps with high kurtosis indicate that there is a small optimal design region for successful insertion that falls rapidly when deviated from in either direction. Lower values of kurtosis suggest that the peak is more spread, and there is no clear delineation between failure and guaranteed insertion. Variance provides a measure of how quickly the likelihood of insertion increases or decreases along changes in a design parameter, which loosely allows comparisons of the importance between the two parameters from the map on insertion potential. Skewness measures whether a parameter positively or negatively influences insertion potential. Distributions with a positive skew (right-tailed) suggest that increasing the design parameter negatively influences insertion potential. Conversely, a negative skew (left-tailed) implies increasing the value of the parameter improves insertion capability. In addition, multi-variable regression analysis was used to quantitatively assess the influence of each parameter on the safety factor.

## 3. Results

### 3.1. Model and Fabrication Comparisons

**Figure 3:**
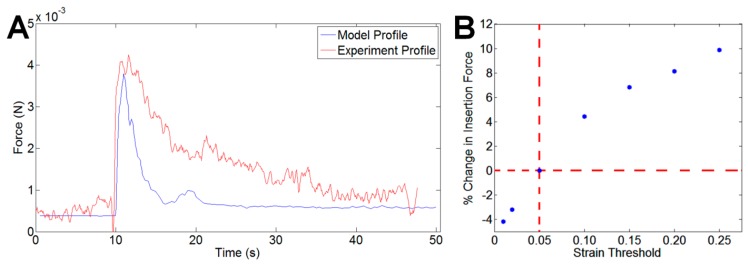
Force profile for insertion phase from (**A**) a representative experiment, and a simulation for a 20 µm × 5 µm SU-8 probe with a 75 µm × 100 µm coating inserted in agarose phantom. Profiles showed the characteristic peak when the probe first penetrates the tissue phantom; (**B**) Changing the strain threshold criterion for element deletion changed the resultant predicted insertion force by up to 10% from the chosen value of 0.05.

Figure 2B,C shows a side-by-side comparison of the simulated coated probe and a scanning electron microscope image of a representative coated probe. Mechanical similarities between simulated and fabricated coated probes were corroborated by the comparable insertion profiles we observed, as indicated in Figure 3A.

### 3.2. Insertion and Buckling Tests

**Figure 4:**
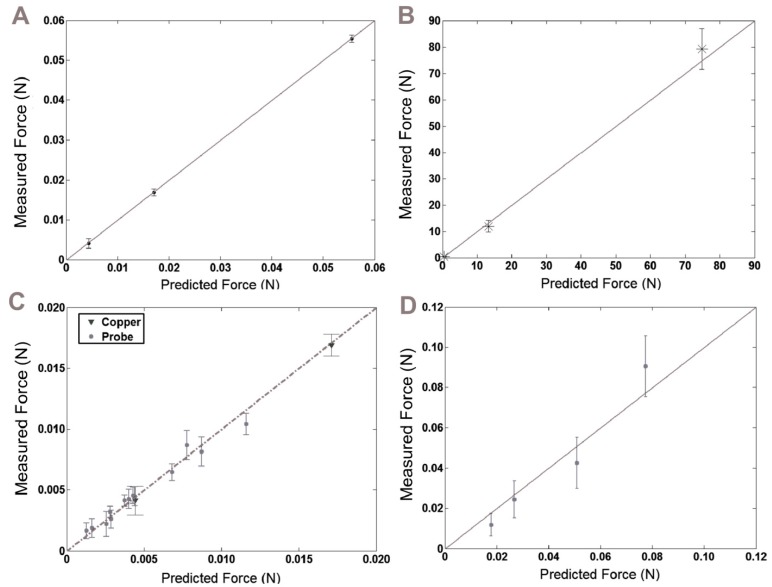
Model accuracy was verified by comparing experimental results to model predictions in same conditions for (**A**) copper wire insertion into 0.6% agarose gel (*R*^2^_insertion_ = 0.967); (**B**) copper wire buckling (*R*^2^_buckling_ = 0.883); (**C**) probe insertion into E18 embryonic chick brain tissue (*R*^2^_insertion_ = 0.975); and (**D**) probe buckling (*R*^2^_buckling_ = 0.878).

Insertion tests with copper wire in agarose were used to validate the FE models of buckling and insertion. Figure 4A,B demonstrates the correlations between measured and model-predicted forces for the insertion and buckling cases, respectively. There was strong agreement between model and experimental results (*R*^2^_insertion_ = 0.975, *R*^2^_buckling_ = 0.878).

The next sets of tests were conducted with a selection of coated and uncoated SU-8 and Parylene C probes in embryonic chick brain tissue. Figure 4C,D shows similar plots of measured force against model predicted forces, demonstrating strong agreement between model and experimental results (*R*^2^_insertion_ = 0.967, *R*^2^_buckling_ = 0.883). Uncoated SU-8 probes successfully inserted for both of the dimensions tested (320 µm × 5 µm and 320 µm × 20 µm). None of the uncoated Parylene C probes successfully inserted. When Parylene C probes were coated, there was a significant reduction in the failure rates. For example, a 50 µm × 50 μm coating on the 20 µm × 5 μm Parylene C probe reduced the failure rate by 50%, and a larger, 50 µm × 100 μm coating reduced it by another 37.5%. Every coated SU-8 probe inserted successfully without buckling.

**Figure 5:**
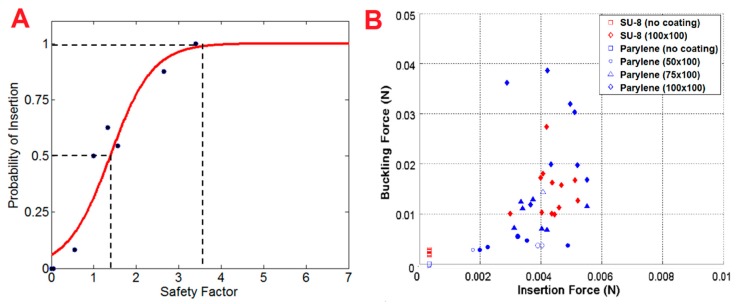
(**A**) Empirical probability of successful insertion *vs.* the “Safety Factor”. Each data point represents the number of successfully inserted probes divided by the total number of probes tested in that particular cohort. The data were fit with a sigmoidal function. A safety factor of ~1.35 corresponds to a 50% likelihood of insertion. A safety factor of ~3.5 corresponds to 100% success rate. (**B**) Buckling force *vs.* insertion force for experiments with rat brain tissue. Filled data points correspond to coated probes that inserted successfully, while empty points correspond to probes that failed (lengths in legend in µm).

Following tests on embryonic chick brain tissue, we selected probe designs for insertion tests in *ex vivo* rat brain based on their likelihood of failure as predicted by simulation results. Figure 5B shows a plot of buckling force *vs.* insertion force for the selection of coated and uncoated probes tested. Similar to the chick brain tests, all of the uncoated Parylene C probes buckled during insertion. A 100 µm × 100 μm coating was the minimum needed to ensure a 100% successful insertion rate, with varying degrees of success with smaller coatings. A 50 µm × 100 μm, which was the smallest coating tested for the Parylene C probes, amounted to a 62.5% success rate.

### 3.3. Model Results

Figure 3 displays a representative plot of force during insertion for a 75 µm × 100 µm coated SU-8 probe from experiment and simulation. Overall, predicted values for insertion forces were within 10.4% of experimental values. Simulated force profiles strongly matched experimental results for insertion tests. Force distributions for each of the models showed that the coating experienced the majority of the load during insertion. Force was primarily distributed on the bottom of the probe and coating once the sample penetrated the tissue. Based on contour plots, the sides of the coating in contact with the tissue experienced frictional forces about an order of magnitude smaller than that experienced at the bottom of the probe and coating. To assess the effect of friction on insertion forces, we adjusted the coefficient of friction from 0 to 0.5 [46]. While there was no appreciable effect on the insertion force (∆F_max_ = +6.2%), frictional force experienced by the sides of the coating increased by 36%. In all cases, the maximum frictional force was still an order of magnitude below the measured insertion force (Frictional force = ~10^−4^ N).

We performed a sensitivity analysis on the hyperelastic parameters that defined the mechanical properties of the tissue in our model by measuring the change in insertion force with each varied parameter. A 10% change in the bulk modulus, *κ*, resulted in the most drastic change to predicted insertion force (from 1.19 mN to 5.54 mN for the 75 µm × 100 μm coating). We also evaluated the influence of our shear strain threshold for element deletion by varying the threshold from 0.01 to 0.25. Our default threshold value was 0.05. With this range of strain threshold values, the insertion forces varied from −4% to +12% of the insertion force from simulations with the default threshold value (Figure 3C).

### 3.4. Model Predictions for Probe Performance

Parallel examination of the insertion and buckling forces from the parametric simulations allowed predictions of probe performance for various conditions. In ideal circumstances, a safety factor above one—*i.e.*, the predicted force required for insertion is less than that required for buckling—would correspond to successful insertion experimentally.

As shown in Figure 5A, we found a strong sigmoidal relationship between probe success rate and the safety factor. We fit a logistic equation to estimate the expected success rate as a function of safety factor, which allowed us to directly link model predictions to the probe performance in an experimental setting.

**Figure 6:**
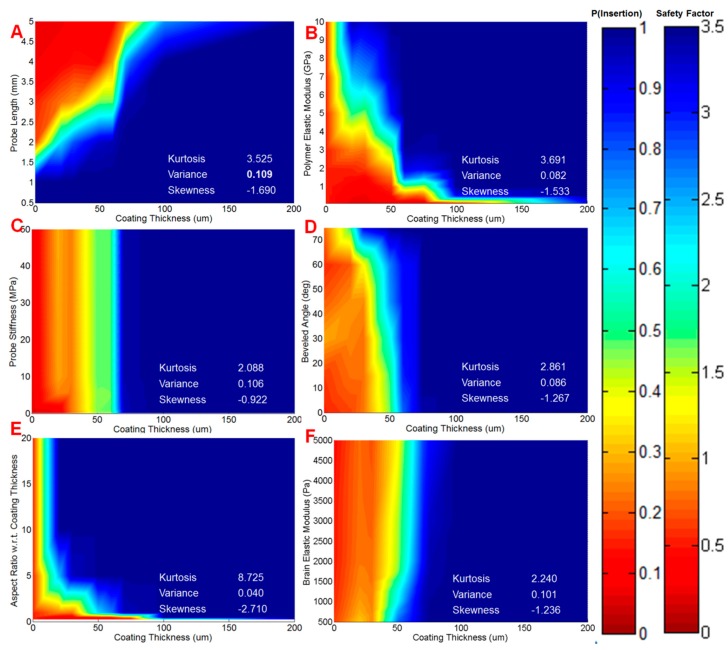
Color maps of the probability of successful insertion of coated probes for six different parameters with respect to coating thickness (0–200 µm): (**A**) probe length; (**B**) polymer stiffness; (**C**) probe stiffness; (**D**) bevel angle of coating tip; (**E**) coating aspect ratio; and (**F**) brain stiffness. To quantitatively assess and compare pairs of parameters in each color map, we calculated the kurtosis, variance, and skewness of each distribution. The largest variance was calculated for probe length *vs.* coating thickness. The largest kurtosis and skewness were calculated for aspect ratio *vs.* coating thickness.

Using this relationship, we assessed the impact of each of the probe specifications on insertion potential with parametric simulations. The range of values for each parameter was based on design constraints of the physical probe and coating procedure: probes could be fabricated with lengths from 0.5 mm to 5 mm, and coated with polymer to a thickness of 0 µm (uncoated) to 350 µm on each side, defining this range as our design space with respect to length and coating thickness, respectively. The safety factor for individual pairs of parameters (defined as *ρ*_1_ and *ρ*_2_) was then plotted against each other to map out how the insertion probability changes with design. Figure 6 depicts probability color maps of how changing coating thickness with other geometric and material parameters affects insertion potential.

The color maps provided a visual representation of insertion probability. To evaluate the importance of the different design parameters and other variables, we calculated the bivariate kurtosis, variance, and skewness of each probability distribution map. Each color map generated a three-dimensional array of values, which prevented the calculation of a single statistic per color map. To determine a single value for each statistic for a given color map, we calculated the kurtosis, variance, and skewness for each 2-D distribution generated along *ρ*_1_, for a fixed value of *ρ*_2_. This was repeated for all the values of *ρ*_2_, and the results were averaged to give a single statistic for a given pair of design parameters. For the regime of coated probes, our comparison of coating aspect ratio *vs.* coating thickness generated the highest kurtosis (*β_kurt_* = 8.73), and skewness (*α_skew_* = −2.71). The color map for this pair of parameters is shown in Figure 6E. Visually, the high kurtosis is represented by the marked delineation between probe failure and successful insertion, which occurs at approximately 20 µm coating thickness. The relatively large value for skewness is represented by the 100% probable insertion predicted in the regions to the right of the narrow transition region. The highest variance calculated was for the comparison of probe length and coating size (σ^2^ = 0.1089).

**Figure 7:**
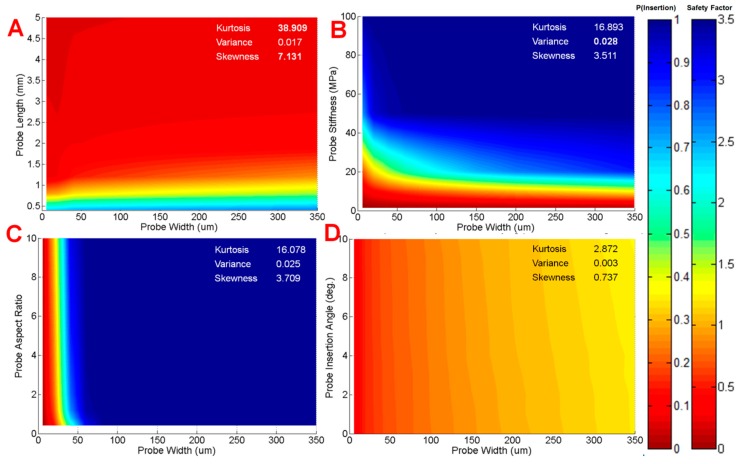
Color maps of the probability of successful insertion of uncoated probes for four different parameters with respect to probe width (0–350 µm): (**A**) probe length; (**B**) probe stiffness; (**C**) probe aspect ratio; and (**D**) probe insertion angle. Color maps demonstrated that the regime of successful insertion fell within unfeasible or ineffectual design ranges, confirming the necessity of the coating for probe insertion into brain tissue. Generally, values for kurtosis and skewness calculated from color maps were greater for uncoated probes than coated probes.

When we included uncoated probes in our analysis, the highest kurtosis and skewness were found to be exhibited with uncoated probe width *vs.* probe length (*β_kurt_* = 38.9, *α_skew_* = 7.13). The minimum length for an uncoated Parylene C probe to reach a reasonable probability of successful insertion was 200 μm, which would not penetrate deep enough into tissue to obtain useful signal. Similar results were seen for other pairs of parameters we plotted, as seen in Figure 7, demonstrating that uncoated probe designs that were predicted to insert into tissue were either outside of the fabrication limits, or surmised to be too big to mitigate the chronic response.

Multi-variable linear regression was used to quantitatively determine the individual effect of each parameter on the safety factor. Results are provided in Table 3 with the design parameters listed in descending order of significance based on the *p* value calculated in our regression model. The safety factor was most dependent on probe length and coating thickness.

## 4. Discussion

We developed and validated a finite element model to design and evaluate the mechanical performance of flexible neural probes that are coated with a polymer to enable insertion into brain tissue without buckling. For a given probe design under ideal, theoretical conditions, when the insertion force exceeds the buckling force, we expect that design to fail. By correlating actual failure rates to the ratio of buckling-to-insertion force, we defined a safety factor that was related to the probability of successful insertion. We are using this design tool to assist in our development of smaller, ultra-flexible probes to minimize trauma but maximize recording area [30].

Experimentally, we used three materials that provided a wide range of mechanical properties and insertion potential to assist in model design: copper, SU-8 photoresist, and Parylene C. Copper is 100 times stiffer than SU-8 and SU-8 is about 1000 times stiffer than Parylene C. Copper specimens inserted successfully at all diameters tested. Uncoated SU-8 probes inserted successfully for sizes as small as 320 μm × 5 μm. Uncoated Parylene C probes failed to insert. When a stiff coating was added to the flexible Parylene C probe, the coating effectively shielded the probe and, if thick enough, allowed insertion before buckling.

The peak force experienced by the probe occurred immediately before penetrating the brain or brain surrogate, assuming that the probe did not buckle, which was captured in our FEM of penetration into brain tissue. Modeling tissue mechanics due to penetration is inherently complex due to soft tissue’s non-linear, anisotropic nature, and the initiation of failure within the tissue, which often results in non-convergent solutions [27]. To model probe penetration into brain tissue, we used element deletion, which removes elements after a user-specified stress or strain threshold is reached. To estimate this threshold, we used results from our experimental validation in the agarose phantom and chick embryo brain tissue to define a preliminary strain threshold. We then adjusted the threshold strain in our simulations until the predicted insertion force generally agreed with experimental values. Our final threshold value fell with the range of failure strains reported in the literature [43,44], including strains measured during insertion of neural electrodes [45].

The insertion force is thus directly dependent on this element deletion criterion, and consequently, so is the safety factor. Using our criterion for element deletion, we found that a safety factor of 3–3.5 corresponded to a probe design which inserted successfully 100% of the time. Lower safety factors corresponded to a lower success rate, and a safety factor of 1.35 indicated a 50/50 ratio of success and failure. Failure properties for CNS tissue vary across the literature, [43,47,48] and our adoption of a threshold of 0.05 should not be viewed as a prediction of the failure properties, but rather a means to an end. If we used a different value for element deletion, the shape of the relationship between the computationally derived safety factor and the experimentally determined probability of successful insertion would not change significantly, although the value for an appropriate safety factor would change.

Our model results were validated against experimental results, with the predicted insertion and buckling forces within 10% of their experimental values for the designs we tested. Values for insertion force from our experiments were in the same order of magnitude as those seen in the Sridharan study [28], which used probes of similar dimensions. The dependence of the buckling on probe properties and dimensions were consistent with Euler’s equation for buckling. As such, for simple geometries under ideal conditions, the insertion force could potentially be estimated analytically by, for instance, modeling a point force on an elastic half space, and then incorporating a stress or strain threshold based on experimental data.

**Figure 8:**
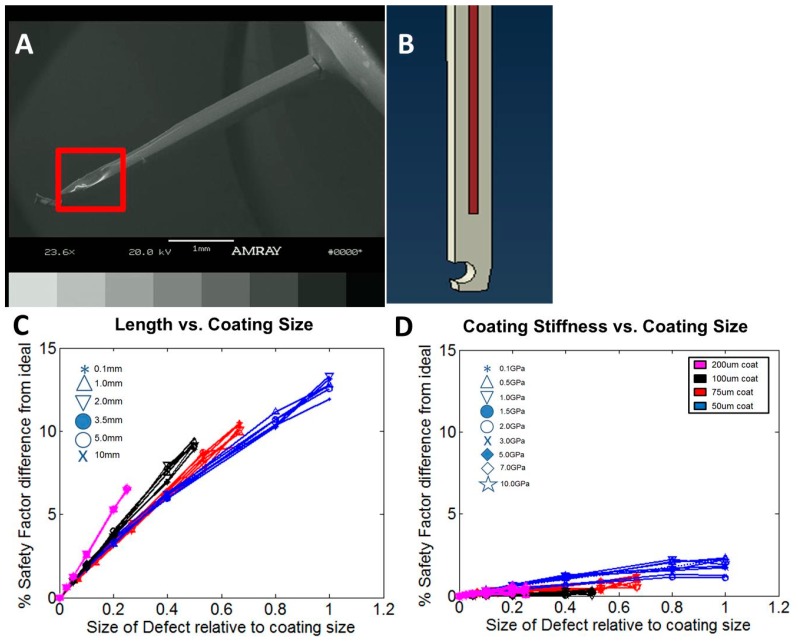
(**A**) Scanning electron microscope (SEM) images of a flawed probe with non-uniform coating. The red box highlights the presence of a divot in the coating. In general, most of these defects occurred at the tip of the probe; (**B**) The FE simulation was modified to mimic the defect we observed in SEM images. The position and size of the defect was varied to determine changes to safety factor. In general, changes in defect size had a greater impact on safety factor than defect position; (**C**,**D**) The percentage difference in safety factor from the ideal (no defect) case with changes in parameter and defect size. The percentage difference in safety factor was plotted against the size of the defect relative to coating size (e.g., 0.25 = 50 μm/200 μm).

However, the likelihood of probe insertion success can be influenced by stress concentrations, positioning errors, and non-uniform geometries. These features can all be easily implemented into the FEM to assess their role in influencing insertion likelihood. For example, we examined the influence of bevel angle on insertion and buckling. The model predicted that a bevel angle of at least 60° is required to appreciably reduce the insertion force. The FEM also allows investigations of design and manufacturing concerns. During testing, some of the coated Parylene C probes that were predicted to succeed based on our model failed to insert. Among the possibilities for this failure include defects in the probe, non-uniform coating distribution, or swelling in the coating due to water resorption. Using our model, we introduced a cylindrical void into the coating to simulate a coating defect and determined the changes to safety factor as a result. Simulation results showed that with a void diameter of 50 μm, the maximum decrease in safety factor was about 15% (Figure 8) when the defect occurred at the beveled tip of the coating. We observed a clear trend where the change in safety factor increased with growing void size. The variation in safety factor with defect size indicates that even at a safety factor of 1.35, where the logistic curve between insertion likelihood and safety factor is steepest, the probability of insertion changes at most ~5%.

Probability color maps, which denoted how the likelihood of insertion varied with changes in pairs of parameters, allowed us to qualitatively assess the influence of each device feature on insertion success. We analyzed eight design parameters in this study, which corresponded to 56 pairs, or 28 color maps. Figure 6 shows probability color maps for a number of parameters compared with coating thickness. Including features such as coating non-uniformity, or different probe geometries would serve to exponentially increase the number of color maps electrode designers would need to review. This provided the rationale to determine how to quantitatively rank the role of each design parameter on safety factor. To quantitatively make this assessment, we used two sets of measures: (1) pair-wise statistical comparisons between parameters; and (2) multi-variable regression. We compared pairs of parameters by calculating the bivariate kurtosis, variance, and skewness of our generated probability surface maps.

We consistently observed that kurtosis was highest for pairs of parameters in uncoated probes compared to coated probes. Figure 7 shows color maps and respective statistics for uncoated probe width plotted against various design parameters. Uncoated and coated probes, which had a high kurtosis, delineated a more sudden transition from a region of failure to success. For instance, in the case of probe length *vs.* uncoated probe width (Figure 7A), there is a clear boundary between 0.2 and 0.8 mm where insertion potential quickly dropped as length increased. In contrast, a low kurtosis, such as Figure 6C (probe stiffness *vs.* coating thickness), indicates a broader “peak”, where the transition from failure to success was gradual. Variance measured how quickly insertion potential increased (or decreased) with parameter change. Coating thickness *vs.* probe length was calculated to produce the highest variance (Figure 6A for color map). This is reflected in the color map by the wide variation in insertion potential, where the upper left quadrant of the color map is primarily red (failure), and the remainder of the map is blue (success). Skewness measured whether the potential for insertion increased or decreased with changes in the pair of parameters. We found skewness was most negative for probe width *vs.* probe length (Figure 7A), suggesting increasing width increased insertion potential.

Individually, each statistic does not reflect the general shape of the color probability map. The high kurtosis we observed for probe length *vs.* probe width, for example, does not indicate whether increasing probe width would increase or decrease insertion capability, nor does it indicate how quickly it would change. When these statistics are taken collectively, however, we can quantify the influence between the pair of parameters on each other, as well as whether the pair has a positive or negative impact on each other, offering the means to rank pairs of design parameters. High kurtosis values calculated for coating aspect ratio *vs.* coating thickness tell designers that past a coating thickness of 30 µm, any increases in aspect ratio will marginally increase the probability of insertion (until it reaches a probability of 1 after which it remains there). The corresponding variance, which was calculated to be lowest in the cohort of coated probes, informs designers that there are no sudden rises or drops in probability, and the strongly negative skew suggests that increasing thickness favors insertion potential.

In contrast to the statistical measures above, which can only be used for pairs of parameters, multi-variable regressions provide a method to assess the role of each design parameter individually against all other parameters. Multi-variable regressions confirmed that probe length and coating thickness have the most significant effects on safety factor (Table 3), and thus should be the first features to consider in design. It is important to note that these regressions and color map analyses are limited to the range of the parameters we examined. We selected the range based on both the limits of the fabrication process for our coated ultra-flexible probes [30], as well as the feasibility of their use. Although an uncoated Parylene C probe 200 µm in length is predicted to insert successfully, it will not be effective in recording because it will not be able to reach target tissues. Increasing the range of values our parameters can take would inevitably change the values predicted by our regression, though we expect trends to be similar (e.g., probe length and coating thickness would still impact safety factor the most).

Although our model successfully simulates and captures insertion potential for different designs of coated probes, it does not predict the specific effects on the chronic response. We are extending our model to predict the interfacial stresses between the already-inserted probe and tissue and how that changes with probe geometry and material, in order to equate stress values with astrocyte activation. With that said, there is good evidence that smaller, flexible neural implants mitigate the acute and chronic injury, and thus, are able to record signals for an extended period of time. Smaller probes reduce the insertion force generated, which theory and our model confirms, and smaller probes have been equated to less tissue damage, and less acute reactive tissue [16]. Flexible probes are predicted to reduce strains experienced by surrounding brain tissue due to micromotions, which have been implicated in the long-term chronic response [17]. Decreasing probe size and stiffness increases the likelihood of probe mechanical failure. However, clinicians may accept the increased chance of failure if it improves the probe’s long-term recording capability. As a hypothetical example, it may be prudent to accept an 80% success rate if it enables the probe to maintain recording fidelity for twice the length of time as the 100% success rate probe. Neural probe designers can utilize our model to design and fabricate probes that fit the criteria, eliminating the guesswork and lengthy fabrication time required to pinpoint those designs which would achieve the necessary result.

## 5. Conclusions

Our work highlights the capabilities of computational modeling to aid in neural electrode design. Compliant probes coated with a stiff, tyrosine-based degrading polymer are designed with the hope that the softer implants will mitigate the chronic response. The vast selection of probe and coating geometric parameters make the use of computational modeling to assess probe performance a prudent design choice. Our validated finite element simulations successfully predict insertion and buckling forces within 10% of their experimental values. From this, we calculated a “safety factor”: the ratio of predicted buckling force and predicted insertion force. We found a relationship linking model-predicted safety factors to empirical probabilities of probe insertions for a variety of geometries, and utilized this relationship to construct probability color maps to qualitatively assess the role of each design parameter on insertion performance. Future work will focus on modeling probe and tissue behavior following insertion to examine the effects of geometry and material properties on the chronic response. The eventual goal is an all-encompassing model that assesses various coated probe designs and the effects on the acute and chronic response for the purposes of optimization.

## Figures and Tables

**Table 1:** Cohorts of probes tested in *ex vivo* chick embryonic brain tissue.

Cohort	Probe Material	Probe Dimensions (μm)	Coating Dimensions (μm)	Total	Pass	Fail	% Success Rate
1	SU-8	320 × 5 × 3500	-	8	8	0	100.0
2	SU-8	320 × 20 × 3500	-	8	8	0	100.0
3	SU-8	320 × 20 × 3500	50 × 50 × 4000	8	8	0	100.0
4	SU-8	320 × 20 × 3500	75 × 100 × 4000	8	8	0	100.0
5	SU-8	320 × 20 × 3500	100 × 100 × 4000	8	8	0	100.0
6	Parylene C	20 × 5 × 3500	-	8	0	8	0.0
7	Parylene C	100 × 5 × 3500	-	8	0	8	0.0
8	Parylene C	320 × 5 × 3500	-	8	0	8	0.0
9	Parylene C	320 × 20 × 3500	-	8	0	8	0.0
10	Parylene C	20 × 5 × 3500	50 × 50 × 4000	8	4	4	50.0
11	Parylene C	20 × 5 × 3500	50 × 100 × 4000	8	7	1	87.5
12	Parylene C	20 × 5 × 3500	75 × 100 × 4000	8	8	0	100.0
13	Parylene C	20 × 5 × 3500	100 × 100 × 4000	8	8	0	100.0
14	Parylene C	20 × 5 × 3500	250 × 100 × 4000	8	8	0	100.0
15	Parylene C	20 × 5 × 3500	350 × 100 × 4000	8	8	0	100.0

**Table 2:** Cohorts of probes tested in *ex vivo* rat brain tissue.

Cohort	Probe Material	Probe Dimensions (μm)	Coating Dimensions (μm)	Total	Pass	Fail	% Success Rate
1	SU-8	40 × 10 × 3500	-	11	6	5	54.5
2	SU-8	40 × 10 × 3500	100 × 100 × 4000	12	12	0	100.0
3	Parylene C	40 × 2.5 × 3500	-	8	0	8	0.0
4	Parylene C	40 × 2.5 × 3500	50 × 100 × 4000	8	5	3	62.5
5	Parylene C	40 × 2.5 × 3500	75 × 100 × 4000	8	7	1	87.5
6	Parylene C	40 × 2.5 × 3500	100 × 100 × 4000	12	12	0	100.0
7	Polymer Shank	-	100 × 100 × 4000	8	8	0	100.0

**Table 3:** Multi-variable regression results for the different parameters modeled in probe designs *versus* safety factor sorted in order of ascending *p* Value. Ranges modeled were selected based on limits of the fabrication process (for geometries), and potential selection of probe materials.

Parameter	Range Modeled	Coefficient Value	95% Confidence Interval	*p* Value
Coating Thickness	0–200 µm	15,513 µm^−1^	[11928, 19113]	<0.0001
Probe Length	0.01–10 mm	−33,037 mm^−1^	[−41029, −26755]	<0.0001
Coating Stiffness	0.001–100 GPa	1857 (GPa)^−1^	[1322, 2274]	<0.0001
Coating Aspect Ratio	0.1–10	5480	[3922, 7034]	<0.0001
Probe Width	5–350 µm	62 µm^−1^	[54, 69]	<0.0001
Beveled Angle	0–75°	1398	[515, 2236]	0.0043
Probe Stiffness	1–5000 MPa	434 (MPa)^−1^	[131, 768]	0.0056
Brain Stiffness	0.5–5 kPa	−25 (kPa)^−1^	[−39, −11]	0.0114

## Supplementary Files

Supplementary File 1
