# Supplementary material for: Modeling the Insertion Mechanics of Flexible Neural Probes Coated with Sacrificial Polymers for Optimizing Probe Design
Source: Sensors (Basel). 2016 Mar 4;16(3):330. doi: 10.3390/s16030330 (PMC4813905; doi:10.3390/s16030330)
Supplement: Supplementary File 1 [file sensors-16-00330-s001.pdf]

Supplementary Materials: Modeling the Insertion Mechanics of Flexible Neural Probes Coated with Sacrificial Polymers for Optimizing Probe Design

Sagar Singh, Meng-Chen Lo, Vinod B. Damodaran, Hilton M. Kaplan, Joachim Kohn, Jeffrey D. Zahn and David I. Shreiber

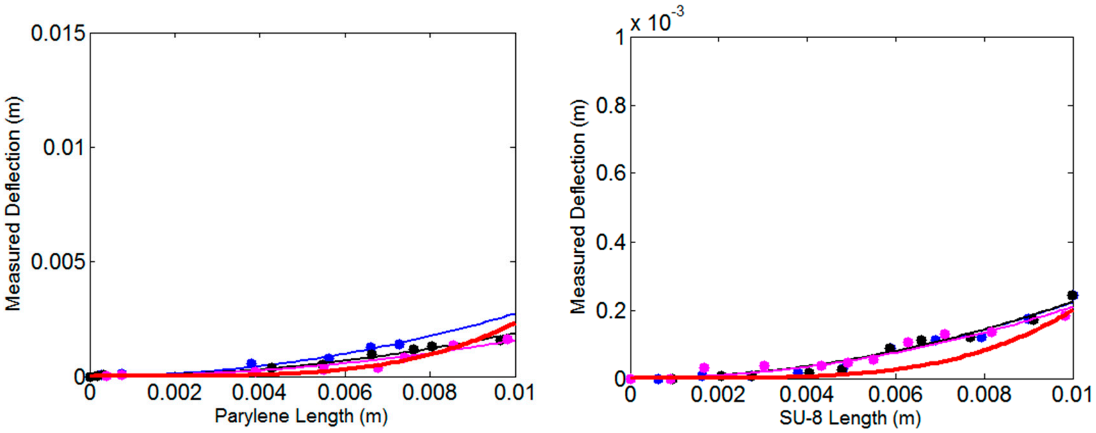

(**a**) (**b**)

Figure S1. Deflection profiles for (**A**) Parylene C and (**B**) SU-8. Curve fitting was used to fit the image-acquired data to the complete beam deflection equation to extract the flexural Young's modulus. The red curve is the resultant fit when the maximum deflection and corresponding flexural modulus ($E_{Parylene}$ = 5.6 MPa, E_{SU-8} = 2.4 GPa) were used.

Table S1. Flexural Young's moduli extracted from deflection tests performed on strips of Parylene C and SU-8 photoresist. To determine the flexural Young's moduli, deflection data measured from images of the freely hanging strips were plotted and fitted with the complete beam deflection equation.

Material (Length)	Flexural Young's Modulus	Correlation Coefficient
Parylene C (1.0 cm × 1.0 cm × 20 μm)	3.39±1.26 MPa	0.854
Parylene C (2.0 cm × 1.0 cm × 20 μm)	5.89±0.58 MPa	0.949
Parylene C (3.0 cm × 1.0 cm × 20 μm)	4.71±0.06 MPa	0.936
SU-8 (0.5 cm × 0.5 cm × 20 μm)	2.10±0.24 GPa	0.985
SU-8 (1.0 cm × 0.5 cm × 20 μm)	1.87±0.33 GPa	0.977
